# A case report of optic neuropathy following dacryocystorhinostomy in a 57-year-old female patient with May-Hegglin anomaly

**DOI:** 10.1186/s12886-020-01433-w

**Published:** 2020-04-19

**Authors:** Seung Uk Lee, Hyoun Do Huh, Hyun Kyung Cho, Su Jin Kim

**Affiliations:** 1 Department of Ophthalmology, School of Medicine, Kosin University, #34 Amnam-dong. Seo-gu, Busan, 602-702 South Korea; 2 Department of Ophthalmology, College of Medicine, Gyeongsang National University, Gyeongsang National University Changwon Hospital, 121, Samjeongja-ro, Changwon, Gyeongsangnam-do 51476 South Korea; 3 Department of Ophthalmology, Pusan National University Yangsan Hospital, Pusan National University School of Medicine, 20, Geumo-ro, Mulgeum-eup, Yangsan-si, Gyeongsangnam-do 50612 South Korea; 4 Research Institute for Convergence of Biomedical Science and Technology, Pusan National University Yangsan Hospital, 20, Geumo-ro, Mulgeum-eup, Yangsan-si, 50612 South Korea

**Keywords:** Dacryocystorhinostomy, May-Hegglin anomaly, Optic neuropathy

## Abstract

**Background:**

We report a rare case of optic neuropathy following dacryocystorhinostomy (DCR) in a 57-year-old female patient with May-Hegglin anomaly.

**Case presentation:**

The patient was presented with sudden onset of vision loss for the left eye after DCR under general anesthesia. Her best corrected visual acuity was light perception in the left eye. Relative afferent pupillary defect was detected in her left eye. Magnetic resonance imaging of the orbit revealed an hyperintensity at the intra-orbital segment of the left optic nerve on T2-weighted image and Flair image. The patient was diagnosed with acute postoperative optic neuropathy and treated with methylprednisolone. Although her vision partially improved, she was left with a visual field defect in the left eye.

**Conclusions:**

In patients with hematologic diseases, postoperative vision loss can occur following even minor surgery under general anesthesia, such as DCR. Therefore, preoperative counseling regarding the risk of visual loss should be given to high-risk patients.

## Background

We report a rare case of an optic neuropathy following dacryocystorhinostomy (DCR) in a 57-year-old female patient with May-Hegglin anomaly. Generally speaking, a DCR surgery is a very common and safe procedure that is conducted successfully throughout the world. In its positive context, a DCR can improve the subjective symptoms and the quality of life of patients with nasolacrimal duct obstructions [1]. However, it is noted that rare complications can occur, including hemorrhage, infection, cerebrospinal fluid leakage, and damage to the medial rectus or superior oblique muscle [2]. Ischemic optic neuropathy (ION) has been documented after a non-complex cataract surgery, uncomplicated pars plana vitrectomy (PPV), and non-ocular surgery, such as with a spinal or cardiac surgery [3–6]. The retinal arterial occlusion (RAO), cortical blindness, acute glaucoma, and choroidal and vitreous hemorrhage have been reported as causes of perioperative vision loss (POVL) after a non-ophthalmic surgery [7–10]. However, the instance of an optic neuropathy following DCR has not yet been reported. Here we report a case of acute postoperative optic neuropathy following DCR in a patient previously diagnosed with May-Hegglin anomaly (MHA).

## Case presentation

A 57-year-old female presented with vision loss in the left eye during the restoration of consciousness after endoscopic DCR surgery for the left eye. In this case, the DCR surgery was performed under general anesthesia. Notably, 2 ml of 1% lidocaine with 1:100,000 epinephrine was injected into the axilla of the middle turbinate and the frontal process of the maxilla using a dental syringe. In this case, the neurosurgical patties soaked in 2 ml of 1:1000 epinephrine were inserted between the inferior turbinate and the nasal septum and in the middle meatus to achieve topical decongestion. In the process of making mucosal flap and incision, the patient had a higher bleeding tendency than was noted with other patients, and a suction diathermy was used meticulously for the incidence of hemostasis. For this reason, it did not lead to a major bleeding in this case.

The patient’s medical history was notable for thrombocytopenia and MHA. Upon review, the patient denied temporal headache, pain, or flashes. When tested, the patient’s best-corrected visual acuity (BCVA) was 20/20 in the right eye and light perception in the left eye. Her intraocular pressure (IOP) was 14 mmHg in the right eye and 16 mmHg in the left eye. Her visual field test result was normal for the right eye. However, the test could not be conducted for the left eye due to the incidence of poor vision. When tested with the swinging flashlight maneuver, a relative afferent pupillary defect was found in the left eye of the patient. Her extraocular movements were noted as being full and painless. However, mild periorbital bruising and swelling were detected in the left eye. Additionally, there was mild maxillary sinusitis noted as well. However, it was shown there was no underlying disease in the other sinuses. On the funduscopic examination, there were no obvious abnormal findings in the macula of either eye. The use of a fluorescent angiography did not reveal leakage or a filling defect at the disc. The baseline testing included blood tests to evaluate syphilis, systemic lupus erythematosus, and neuromyelitis optica. Her erythrocyte sedimentation rate and C-reactive protein results were noted as normal. Her pre-operative platelet count was 61 × 10^3^/mm^3^. A chest x-ray was performed to evaluate sarcoidosis. She was transfused with six units of platelets preoperatively, which increased her platelet count to 123 × 10^3^/mm^3^. No other cause of optic neuropathy was found in this evaluation.

**Fig. 1:**
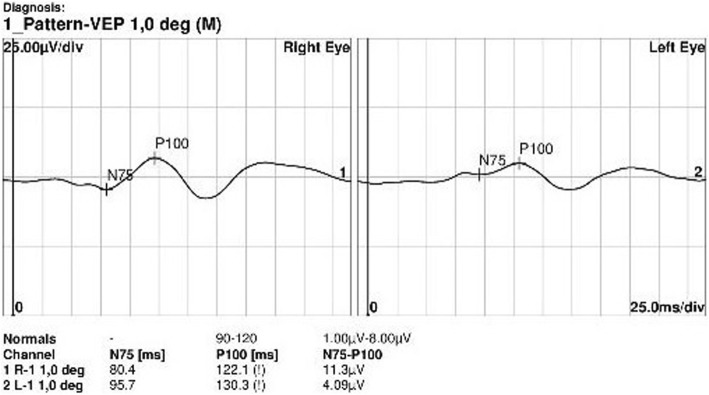
Pattern visual evoked potential (VEP) revealing delay of P100 latency and decreased amplitude

**Fig. 2:**
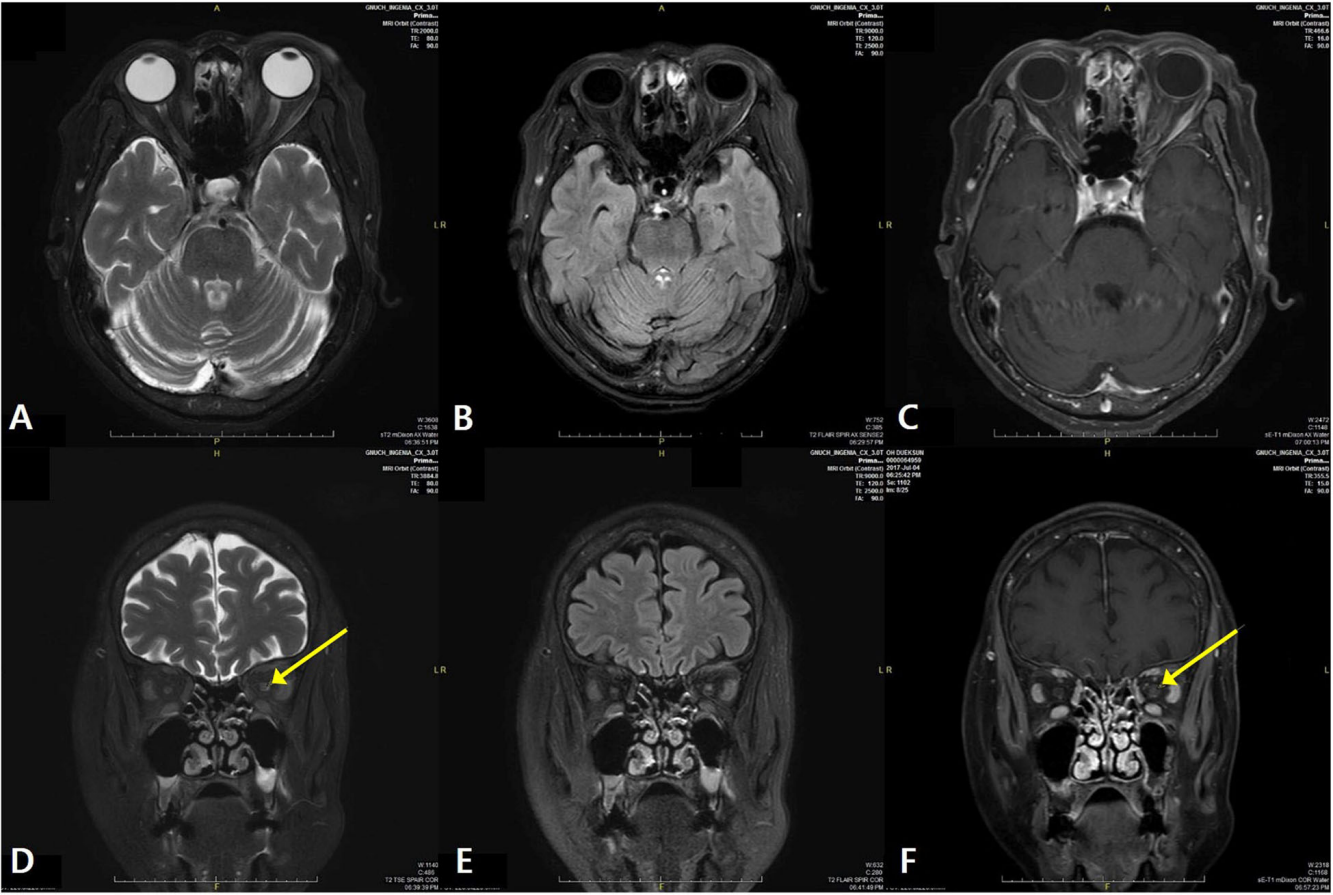
Axial images of brain MRI (**a, b** and **c**: T2-weighted image (T2-WI), Flair images, and T1 post contrast images, respectively). Coronal images of brain MRI (**d, e** and **f**: T2-WI, Flair images, and T1 post contrast images, respectively). T2-WI (**d**) revealing hyperintensity in the intra-orbital segment of the left optic nerve (arrow). T1 post contrast images (**f**) revealing enhancement in the same level (arrow)

The pattern visual evoked potential revealed delayed P100 latency (Fig. 1). Her electroretinogram showed normal electrical activity in the retina. The magnetic resonance imaging (MRI) of the orbit revealed a focal hyperintensity within the intra-orbital segment of the left optic nerve on the T2-weighted image (T2-WI) and flair image. At evaluation, the MRI showed an enhancement on the T1 post-contrast imaging (Fig. 2). It did not show any demyelinating disease in the brain. The patient was diagnosed with left optic neuropathy and treated with 1 g/day of intravenous methylprednisolone for 3 days, followed by 1 mg/kg/day of oral prednisone with subsequent dose tapering. It is noted that the patient’s BCVA improved to 20/30 after the treatment. Although her vision improved, she was left with a visual field defect in the left eye.

## Discussion and conclusions

Perioperative vision loss (POVL) is a rare but devastating consequence of ocular or non-ocular surgery. To this end, the nonarteritic anterior ischemic optic neuropathy (NAION) after cataract surgery or PPV has been reported to result from increased intraocular pressure (IOP), raised intra-orbital pressure from a retrobulbar anesthetic, face-down position, systemic peri-operative hypotension, or a combination of these factors [3, 4, 11, 12]. For this reason, the causes of visual impairment in patients undergoing non-ocular surgery under general anesthesia can be categorized into three main types: ION, retinal vascular occlusion, and cortical vision loss due to a perioperative stroke. The particularly high incidence of ION after coronary artery bypass grafts might be due to an increased blood viscosity caused by induced hypothermia, leading to a watershed injury to the optic nerve head [13–15]. The IOP may also play a role in these cases, because IOP spikes have been demonstrated when bypass circulation begins and the IOP may remain elevated for days after surgery. ION following spinal fusion is most commonly associated with posterior ischemic optic neuropathy (PION) [5]. Ischemia in PION involves the portion of the optic nerve perfused by the small vessels of a pial capillary plexus between the orbital apex posteriorly and the point at which the central retinal artery enters the nerve at its mid-point. As noted, prone positioning can decrease venous outflow by increasing intra-abdominal and intrathoracic pressure, raise IOP, and decrease perfusion pressure of the optic nerve head [16].

In the present case, the patient was diagnosed with MHA, a rare hematological disorder. MHA is characterized by various degrees of thrombocytopenia, giant platelets, and basophilic, cytoplasmic inclusion bodies in the granulocytes [17, 18]. Here, thrombocytopenia occurs in approximately 50% of the patients with MHA. Likewise, the clinical manifestations vary and range from mild bleeding not requiring specific treatment to severe bleeding episodes following trauma or surgery that require the administration of blood products [17, 18]. To the author’s knowledge, there are no guidelines for preoperative prophylaxis in MHA patients. In general, a platelet count of ≥50 × 10^9^/L is recommended for safe procedures [18]. The present patient experienced neither spontaneous hemorrhage nor other complications before. She was transfused with platelets before the DCR surgery to prevent complications which were associated with bleeding.

The possible differential diagnoses of this patient included traumatic optic neuropathy, compressive optic neuropathy, inflammatory optic neuropathy, anterior ischemic optic neuropathy (AION), and PION. During the surgery, there was more bleeding tendency than expected, but hemostasis was well done, in order that there was not much bleeding and there were no other problems noted. Since the pupillary reaction was observed during operation, we could rule out direct trauma to the optic nerve by needle. Additionally, as the IOP may be elevated as the injected local anesthetic material passes to the retrobulbar area, or retrobulbar bleeding occurs by needle injury, or the eyeball was pressed inadvertently by the surgeon or assistant, this may play a factor or a role in the occurring AION. However, on funduscopic examination, there was no optic disc edema or peripapillary hemorrhage. The patient’s visual acuity was improved with methylprednisolone. Thus, the possibility of an ION is low. The optic nerve may have been damaged by adrenaline-induced vasospasm, but this is less likely because the concentration and amount of adrenaline were used the same as usual surgery. Bleeding during surgery might have temporarily resulted in hypoperfusion and ischemia to the optic nerve head. Also, the existence of a PION due to hypotension seems to be the most likely cause of this event.

In this case, despite pre-operative platelet transfusion and uncomplicated surgery, the patient developed vision loss, presumably due to posterior ischemic optic neuropathy. Her underlying hematologic abnormality might have increased the risk of bleeding and caused optic neuropathy. In patients with hematologic diseases, postoperative vision loss can occur following even minor surgery under general anesthesia, such as a DCR. Therefore, preoperative counseling regarding the risk of POVL should be given to high-risk patients, and appropriate prevention should be conducted to minimize the advent of any unanticipated events that can cause devastating visual morbidity.

## Data Availability

All data have been presented within the manuscript and in the form of images.

## Abbreviations

DCR: Dacryocystorhinostomy
ION: Ischemic optic neuropathy
PPV: Pars plana vitrectomy
RAO: Retinal arterial occlusion
POVL: Perioperative vision loss
MHA: May-hegglin anomaly
BCVA: Best-corrected visual acuity
IOP: Intraocular pressure
MRI: Magnetic resonance imaging
NAION: Nonarteritic anterior ischemic optic neuropath
PION: Posterior ischemic optic neuropathy
AION: Anterior ischemic optic neuropathy
